# Supplementary material for: The interactome of intact mitochondria by cross-linking mass spectrometry provides evidence for coexisting respiratory supercomplexes
Source: Mol Cell Proteomics. 2017 Dec 8;17(2):216–32. doi: 10.1074/mcp.RA117.000470 (PMC5795388; doi:10.1074/mcp.RA117.000470)
Supplement: Supplemental Data [file supp_RA117.000470_133922_0_supp_23978_fzffwf.zip › spectra_annotation/mito_spectra_annotation/169-1-9-1-13-1.pdf]

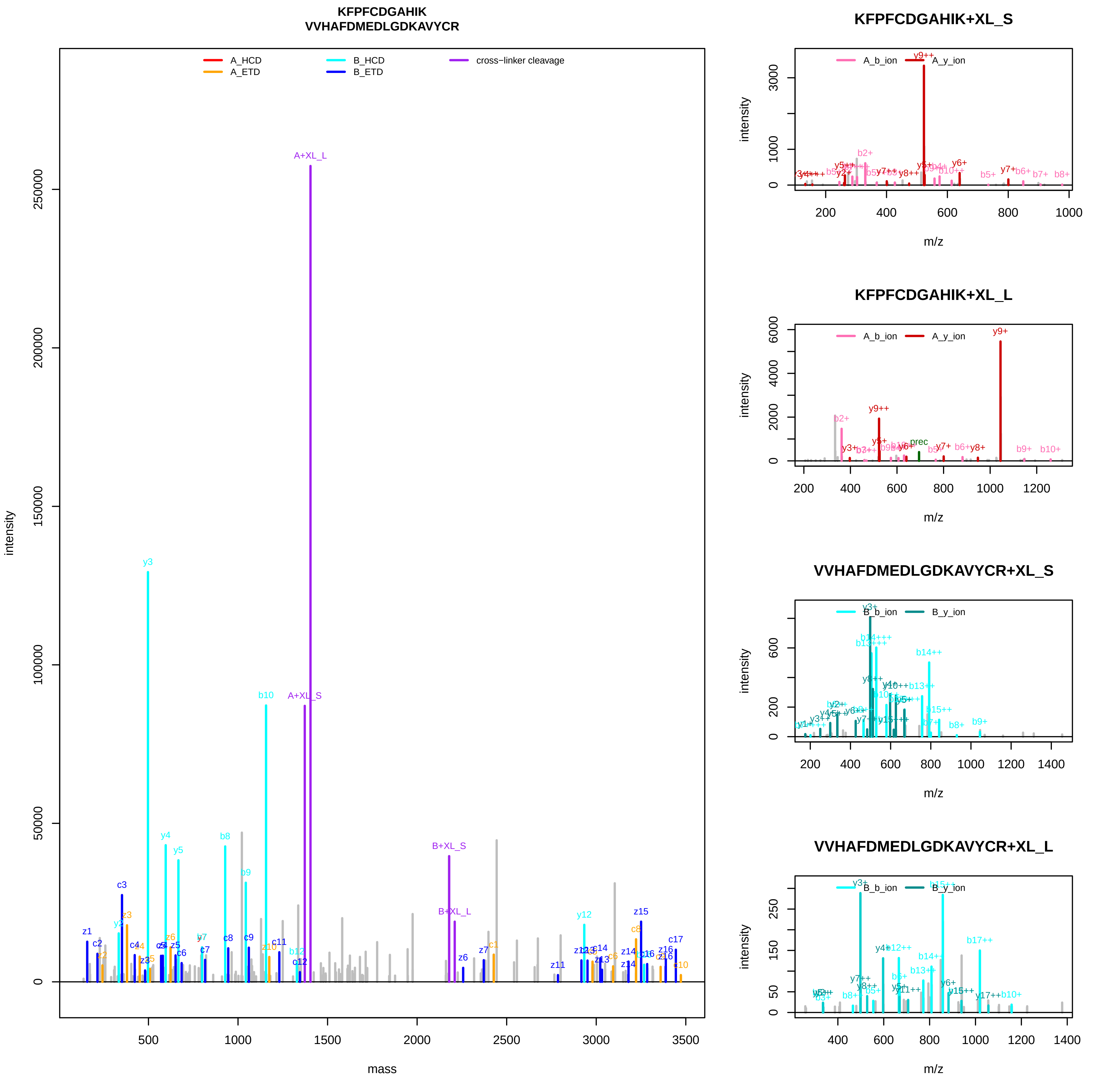

KFPFCDGAHIK
VVHAFDMEDLGDKAVYCR
A_HCD
A_ETD
B_HCD
B_ETD
cross−linker cleavage
A+XL_L
A+XL_S
B+XL_S
B+XL_L
y3
b10
y4
y5
b8
b9
c3
y12
z15
intensity
mass
0
50000
100000
150000
200000
250000
500
1000
1500
2000
2500
3000
3500
KFPFCDGAHIK+XL_S
A_b_ion
A_y_ion
y9++
intensity
m/z
KFPFCDGAHIK+XL_L
y9+
y9++
prec
VVHAFDMEDLGDKAVYCR+XL_S
B_b_ion
B_y_ion
y3+
b14++
VVHAFDMEDLGDKAVYCR+XL_L
b15++
b17++
